# Application of Anesthetics in Cancer Patients: Reviewing Current Existing Link With Tumor Recurrence

**DOI:** 10.3389/fonc.2022.759057

**Published:** 2022-02-28

**Authors:** Xiaotian Liu, Qian Wang

**Affiliations:** Department of Anesthesiology, Children’s Hospital of Soochow University, Suzhou, China

**Keywords:** anesthesia, cancer, tumor recurrence, perioperative factors, inhalational anesthetic, intravenous anesthetic

## Abstract

Surgery remains the most effective cancer treatment, but residual disease in the form of scattered micro-metastases and tumor cells is usually unavoidable. Whether minimal residual disease results in clinical metastases is a function of host defense and tumor survival and growth. The much interesting intersection of anesthesiology and immunology has drawn increasing clinical interest, particularly, the existing concern of the possibility that the perioperative and intraoperative anesthetic care of the surgical oncology patient could meaningfully influence tumor recurrence. This paper examines current data, including recent large clinical trials to determine whether the current level of evidence warrants a change in practice. Available pieces of evidence from clinical studies are particularly limited, largely retrospective, smaller sample size, and often contradictory, causing several questions and providing few answers. Recent randomized controlled clinical trials, including the largest study (NCT00418457), report no difference in cancer recurrence between regional and general anesthesia after potentially curative surgery. Until further evidence strongly implicates anesthesia in future clinical trials, clinicians may continue to choose the optimum anesthetic-analgesic agents and techniques in consultation with their cancer patients, based on their expertise and current best practice.

## Introduction

Cancer constitutes an enormous burden on society in both poor and rich global economies alike. Factors contributing to the increasing occurrence of cancer include the growth and aging of the population, as well as an increasing prevalence of established risk factors such as smoking, physical inactivity, overweight, and changing reproductive patterns associated with urbanization and economic development (1). Some of the most common cancers contributing to high mortality include malignant tumors of the lung, breast, prostate, and colorectum. Surgical removal of malignant tumors remains the primary and most effective treatment option for cancer; however, the surgical procedure results in a significant systemic release of tumor cells (2). The potential of these cells to lead to metastases is largely dependent on the balance between the resilience of the body’s immunity and the aggressiveness of tumor cells (2). Several factors including surgical stress, anesthetic agents, and opioid analgesics can compromise immune function and might shift the balance towards the progression of minimal residual disease.

Metastatic disease is the most important cause of cancer‐related death in patients after malignant tumor surgery (3). The hypothesis that anesthesia may influence cancer recurrence after surgical removal was first proposed in 2006 (4) and has since gained traction as one of the most important research questions in this field (5). In recent years, many studies have investigated the rate of tumor resurgence regarding the different anesthesia techniques and agents, and the significance of anti-inflammatory, anti-cancer, and anti-metastatic effects in the context of anesthesia, providing insights into potential mechanisms by which anesthesia might influence malignant cells. This review examines recent experimental, preclinical, and clinical studies of the different types and techniques of anesthesia used during cancer surgery regarding their influence on the long‐term survival or rate of tumor recurrence in patients undergoing cancer surgery.

## Anesthesia in Cancer Patients

**Figure 1:**
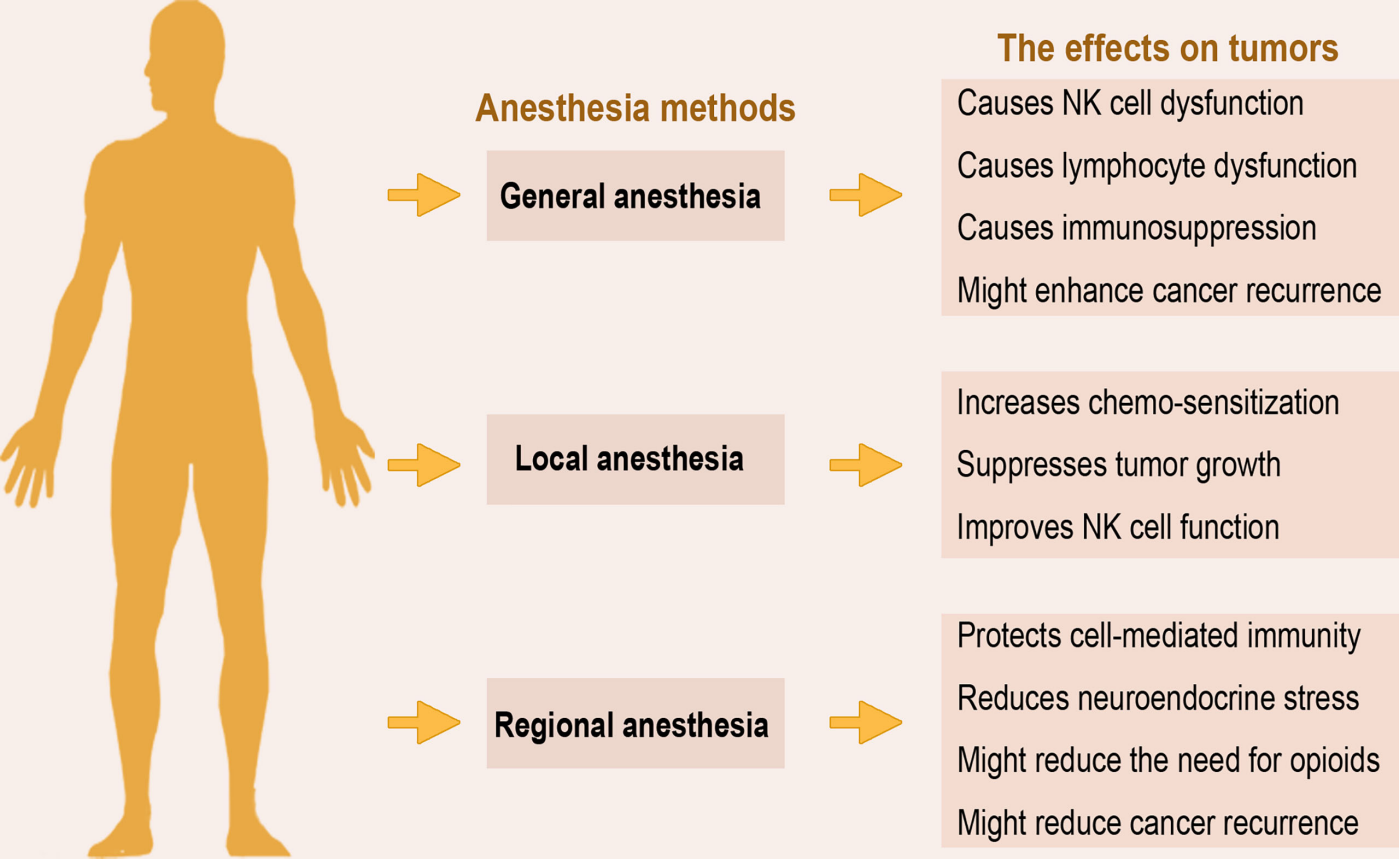
Anesthesia methods and overview of their effects on tumors. The three main anesthesia methods applied in surgery exert varying effects on the host’s immunity and ability to clear residual tumor cells. The overview of current data from animal models, *in vitro*, and human studies, suggests that regional anesthesia may be more preferred to general anesthesia due to its immunoprotective effects.

The perioperative use of anesthesia forms a crucial part of daily clinical practice in patients undergoing surgery. In cancer surgery, the perioperative period constitutes an important stage for the further course of the disease, as circulating tumor cells shed from the primary tumor into the patient’s bloodstream might form new micro-metastases independent of complete tumor removal (6). Various studies have investigated the potential beneficial effect or otherwise of the different anesthesia techniques regarding outcome (overall and/or recurrence-free survival) in patients undergoing cancer surgery. **Figure 1** presents the three main anesthesia techniques employed in tumor surgery and an overview of their effects as discussed below.

### Local Anesthesia

Local anesthesia is employed to numb a small part of the body when surgery is minor and does not require general or regional anesthesia. Local anesthetics are common medication and a mainstay of anesthesia since the introduction of cocaine in 1884 and are administered systemically or used as part of regional anesthesia techniques for a variety of reasons. They are effective in pain relief due to their ability to block the voltage-gated sodium channel, thus inhibiting nerve cell depolarization (7, 8), and may contribute to reducing postoperative nausea and vomiting (9) and enhancing early recovery after surgery (10). Local anesthetics may exert a certain degree of influence on circulating tumor cells shed during surgery through direct or indirect means because of their strong anti-inflammatory properties. For example, they could contribute to blunting the inflammatory stress response induced by the surgical stimulus (6).

On the other hand, certain local anesthetics have been demonstrated to preserve immune cell function and exhibit anti-metastatic effects. They can reduce the viability and proliferation of cancer cells *in vitro*, and efficient to target residual disease or cells that form micro-metastasis. Lidocaine, one of the most applied local anesthetics in clinical settings, has been shown to exhibit multi-activities, including the potential in cancer therapy. Growing evidence shows that lidocaine might not only work as a chemosensitizer that induces other conventional chemotherapies to eliminate certain resistant cancer cells but could also suppress cancer cell growth by single-use at different doses or concentrations (11). *In vitro* studies show that lidocaine improves the activity of NK cells and the intravenous administration of lidocaine as part of the perioperative anesthesia regimen, bears the potential to reduce the risk of cancer progression or recurrence in patients undergoing cancer surgery (12).

### Regional Anesthesia

Regional anesthesia is applied to block pain in a particular region of the body. Some studies have asserted that regional anesthesia methods provide perioperative pain relief, hence reduce the number of systemic anesthetic agents and opioids administered (13). Epidural anesthesia, a form of regional anesthesia, blocks the nerve impulses from the lower spinal segments to induce analgesia or pain relief. In epidural anesthesia, one or more drugs are injected into the epidural space bordering on the spinal dura mater to induce a “central” and/or “neuraxial” block (14, 15). Surgical operations carried under general anesthesia result in the bombardment of the central nervous system with nociceptive input and responses, with a neurohumoral stress response that stimulates the sympathetic nervous system and hypothalamic-pituitary axis. The use of regional anesthesia *via* blockade of nociceptive afferents might inhibit much of this neurohumoral response and its subsequent impact on the immune system. In animal studies, the addition of spinal anesthesia to a halothane anesthetic (16) and sevoflurane anesthetic (17), preserved the immune response and reduced hepatic metastases of tumor cells, while preserving liver mononuclear cell function, and attenuating the downward shift in T helper 1/T helper 2 cytokine balance.

Preclinical and retrospective studies highlight a potential benefit of regional anesthesia as it protects cell-mediated immunity and reduces the surgical neuroendocrine stress response by blocking afferent neural transmission that stimulates the hypothalamic-pituitary-adrenal axis and sympathetic nervous system, hence reducing the need for opioids and volatile anesthetics and therefore reducing cancer recurrence (18, 19). The administration of regional anesthesia results in reduced use of certain anesthesia and pain medications that are given intravenously or inhaled into the lung, and as well attenuate surgical stress (13). Therefore, many studies have suggested that regional anesthesia might reduce the risk of long‐term cancer recurrence.

### General Anesthesia

General anesthesia is a combination of medications that put a patient in a sleep-like or unconscious state and inactivates response to pain signals or reflexes of the autonomic nervous system before surgery. It uses intravenous anesthetics, inhalational (volatile gasses) anesthetics or a combination of both. Opioids and benzodiazepines are often employed as adjuvants during general anesthesia (20, 21). The most frequently applied method in general anesthesia is intravenous anesthesia and uses anesthetic agents such as propofol, sodium thiopental, and ketamine. Volatile anesthetics often used to induce and maintain general anesthesia include sevoflurane, isoflurane, and desflurane. There is evidence that these two general anesthesia methods influence the immune system *via* cellular and molecular (cytokine) modulation, or activation of the hypothalamic-pituitary-adrenal axis and the sympathetic nervous system, and possibly contribute to long-term tumor recurrence after surgical intervention (22–24).

Concerning cancer patients, the immunosuppression associated with general anesthesia, including the dysfunction of natural killer (NK) cells and lymphocytes, could promote the immune evasion, growth, and metastasis of residual cancer cells, hence worsening patients’ prognoses (25, 26). For example, volatile anesthetics have varying influence on immunity through their effects on components such as NK cells, neutrophils, dendritic cells (DCs), and macrophages (25), and inhibit cytokine release, reduce lymphocyte proliferation, trigger lymphocyte apoptosis, and inhibit the function of neutrophils in a dose-dependent manner (27). In a controlled trial, patients undergoing elective reconstructive surgery for tongue cancer were randomized to receive general anesthesia of either propofol induction and maintenance, sevoflurane induction and maintenance, or propofol induction and sevoflurane maintenance (mixed). Results showed that NK cells, B lymphocytes, and T lymphocyte subsets such as CD3(+) cells, CD3(+)CD4(+) cells, and CD4(+)/CD8(+) ratio significantly reduced in all groups. However, further analysis indicated that propofol had slightly less effect on cellular immune responses than sevoflurane (28). These studies indicate the immunosuppressive effects of anesthesia on host immunity, a possible promoter of tumor recurrence.

## Perioperative Factors Associated With Cancer Promotion

### Anesthetics

Perioperative anesthesia and analgesia exacerbate immunosuppression in the already immunocompromised cancer microenvironment in patients. NK cells are a critical part of anti-tumor immunity and are responsible for the phenomenon of immune surveillance, which includes the detection of circulating tumor cells (29). However, the innate immune system, especially NK cell activity is known to be significantly impaired by certain anesthetic agents such as sevoflurane-fentanyl (30). Local anesthetics, especially the amide anesthetics, possess strong anti-inflammatory ability through their effects on cells of the immune system, as well as on others such as microorganisms, thrombocytes, and erythrocytes, which have been extensively studied (31, 32). Although there are different effects regarding volatile anesthetic agents on cancer promotion, the majority of *in vitro* studies suggest that these agents are associated with elevated expression of tumorigenic markers, and increased migration and proliferation of cancer cells (33, 34). For example, enflurane and halothane reversibly and dose-dependently impair NK cell function, and isoflurane and halothane prevent interferon-stimulated NK cell activities (35–37).

Volatile anesthetics not only cause immune cell dysfunction but apoptosis of neutrophils and T-lymphocytes (38, 39), as sevoflurane, one of the most commonly used inhalation anesthetics, induces apoptosis and oxidative stress in lymphocytes (40). In another study, although there were no significant differences in tumor size or survival between sevoflurane and control mice, *in vitro* study showed that the proliferation of Lewis lung carcinoma cells exposed to sevoflurane increased by 9.2% compared to the controls (41). This implies that sevoflurane exposure might enhance the proliferation of tumor cells *in vitro* environment, but might not affect proliferation *in vivo*, suggesting that the effects of anesthetics on *in vitro* studies of cancer do not necessarily translate into *in vivo* or clinical studies.

**Figure 2:**
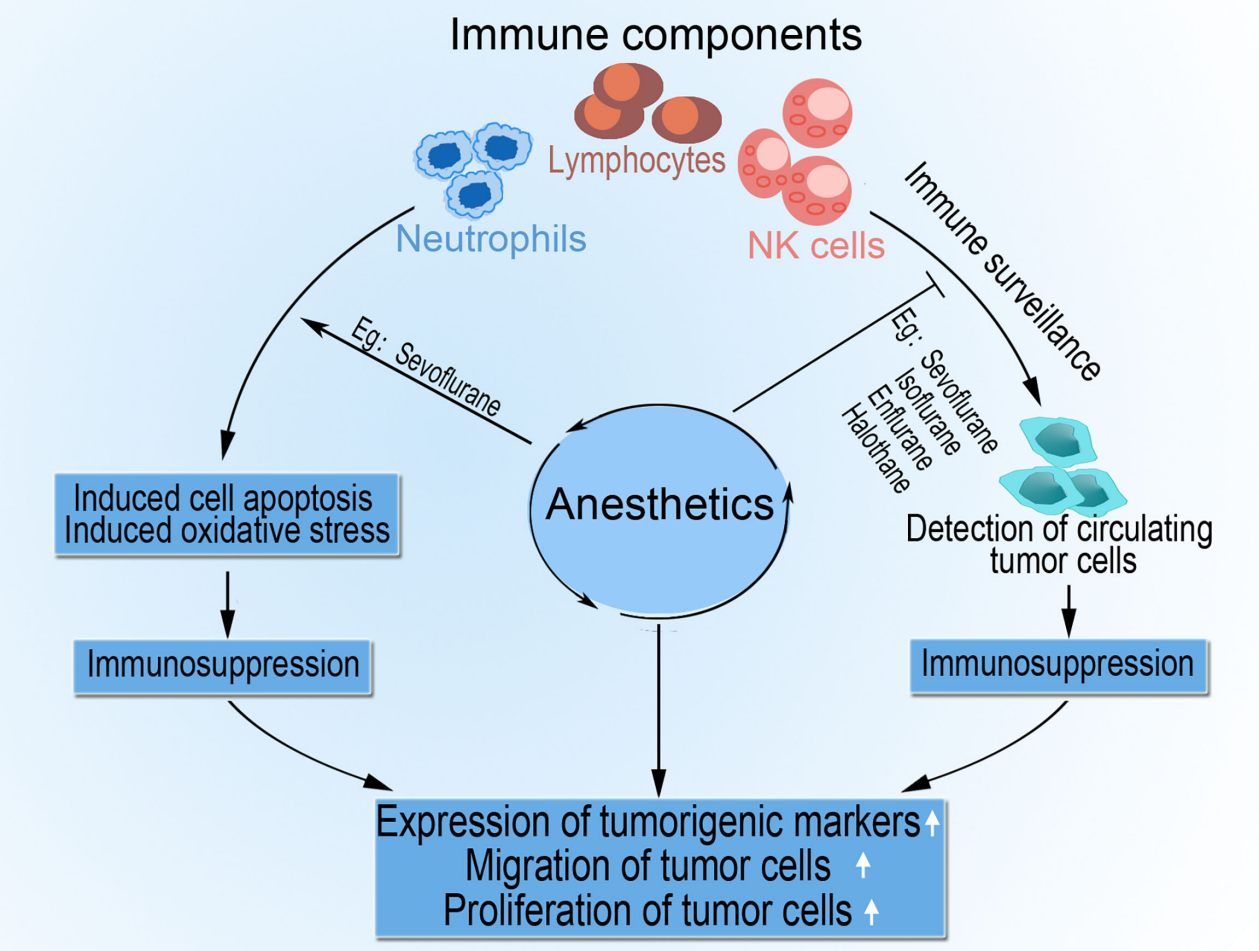
The role of anesthesia in tumor progression. Anesthetic agents impair cell-mediated immunity by direct or indirect inhibition of components such as NK cells, lymphocytes, and neutrophils. Anesthesia also impedes immune surveillance of circulating tumor cells by NK cells and activates apoptosis and oxidative stress in lymphocytes and neutrophils. The resultant immunosuppression encourages tumor cell migration, proliferation, and upregulated expression of tumorigenic markers.

The administration of general anesthesia alone is known to impair immune function; however, the addition of pectoral nerve II block under general anesthesia increases the proportion of NK cells, improves tumor cell killing activity, and upregulates postoperative IL-2 concentration in patients’ plasma (42). Ketamine, a dissociative anesthetic agent with excellent analgesic properties and a favorable safety profile, effectively reduces postoperative pain, blunts hyperalgesia, lowers opiate consumption, and even decreases chronic persistent postoperative pain (43, 44). However, ketamine has tumor modulatory and anti-inflammatory effects, including, promoting tumor growth *via* decreasing NK cells and increasing tumor cell retention (35) and generally inducing immunosuppression (45). **Figure 2** summarizes the complex immunosuppressive effects of anesthesia that aid tumor progression.

### Opioid Analgesics

Opioid analgesics are well-known inhibitors of both cellular and humoral immunity (46, 47). Their effects are primarily modulated by the µ-opioid receptor (MOR) as demonstrated in the evidence that MOR-deficient mice do not exhibit immunosuppression with morphine, and that naloxone blocks morphine-related immunosuppression (36, 48). Morphine has both tumor growth-promoting and -inhibiting effects as reported in many studies (49, 50). In its tumor-promoting influence, morphine stimulates angiogenesis to enhance cancer progression. In one of such studies, the effect of morphine on tumor onset, development, and survival of animal models, as well as whether MOR, mast cell stimulation, lymphangiogenesis, and substance P (SP) are linked with tumor-enhancing effects of morphine was investigated. The outcome indicates that, although morphine does not influence the onset of tumor development, it significantly enhances the growth of existing tumors, and decreases overall survival in mice. The activation of mast cells by morphine may participate in increasing SP and cytokine levels, resulting in cancer progression, while MOR might be linked with morphine-induced cancer progression (51). On the other hand, morphine was shown to inhibit the migration of tumor-infiltrating leukocytes and suppress angiogenesis associated with tumor growth in mice (52).

In general, reports on the effects of opioids on tumor cell migration, proliferation, and apoptosis are contradictory and appear to reflect the influence of multiple factors of tumor biology and drug administration. In these reports, tumor growth either decreases, increases, or remains unaffected by opioid analgesics.

### Surgical Stress

**Figure 3:**
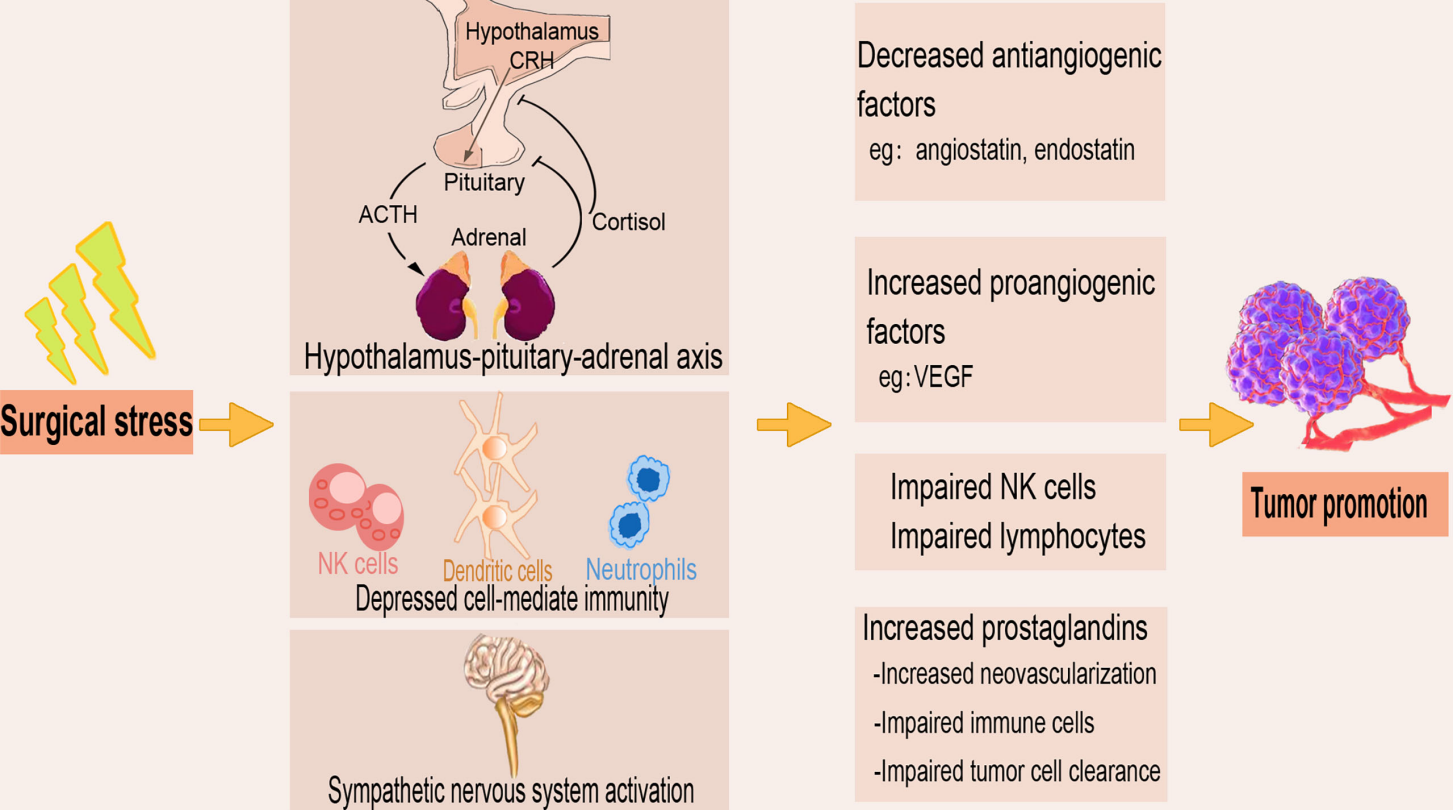
The tumor-promoting effect of surgical stress. The stress produced during the surgical removal of tumors activates the sympathetic nervous system and the hypothalamus-pituitary-adrenal axis and as well depresses the cell-mediated immunity. Surgical stress also decreases antiangiogenic factors, increases proangiogenic factors, and upregulates prostaglandins, leading to impaired immune cell function and tumor cell clearance.

The surgical removal of tumors induces stress which results in depressed cell-mediated immunity and decreased concentrations of tumor-associated antiangiogenic factors such as angiostatin and endostatin (**Figure 3**). The surgically induced suppression of cell-mediated immunity is a summation of both direct cell-mediated influence and indirect paracrine-mediated effects *via* dysregulation of cytokine signaling. Surgery or anesthesia-induced activation of the hypothalamic-pituitary-adrenal axis and the sympathetic nervous system provides immunosuppression through several soluble factors (33, 53). Surgical stress upregulates the concentration of proangiogenic factors, including vascular endothelial growth factor (VEGF), and triggers the release of growth factors that promote local and distant growth of malignant tissue (54, 55). Innate immune components such as NK cells play a crucial role in eliminating circulating tumor cells and preventing metastasis (56), where reduced expression of circulating NK cell phenotypes are associated with tumor progression (57). Many studies report reduced postoperative NK cell and certain lymphocyte subsets functions, and an inverse correlation of NK cell function with tumor stage and metastatic growth (58, 59).

The robustness of an individual’s perioperative cell-mediated immunity plays an important function in postoperative cancer outcomes. In other words, the oncologic outcome after surgery does not only depends on the extent, invasiveness, and type of cancer but the level of the patient’s perioperative immune status and function (60). Cytokines such as interleukins, interferons, and tumor necrosis factors, among other chemical mediators, constitute a complex signaling network that modulates the diverse and interdependent immune cells. In addition to NK cells, other primary effector cells such as macrophages, and adaptive immune system cytotoxic lymphocytes play crucial roles in the tumor outcome (61, 62). In addition to the prostaglandins expressed in abundance due to surgical trauma, tumor cells also produce prostaglandins that together alter the tumor microenvironment, enhance neovascularization, and impair immune cells, adversely affecting the capability to clear residual disease after cancer surgery (63–65).

## Anesthesia and Tumor Recurrence

Following the hypothesis that anesthetic and analgesic techniques during cancer surgery influence recurrence or metastasis, the first set of original investigations and a short overview encompassing a consensus statement were published to highlight concerns and drive more investigations (5, 66). These investigations sought to examine the direct effects of anesthetic and analgesic drugs on cancer cell biology, the effect of anesthetic technique in randomized cancer surgery patients on perioperative host immunity and cancer metastatic function, and new retrospective clinical data on perioperative factors associated with subsequent recurrence or metastasis. Recently, several clinical trials have also been published. While volatile anesthetics and opioids generally suppress cell-mediated immunity and enhance the proliferation of cancer cells and angiogenesis, propofol appears to rather support cell-mediated immunity and inhibit tumor angiogenesis (33).

### Preclinical Trial Studies

Studies on the effects of anesthesia on tumor cells differ depending on the type and technique employed. While some anesthetic agents enhance tumor cell survival, others inhibit their progression. Anesthetic agents vary in their capability to trigger immunomodulation and potentiation of tumorigenic growth factors, including hypoxia-inducible factor-1 (HIF-1α) and insulin-like growth factors (67–69). Reports indicate that isoflurane enhances the malignant potential of ovarian cancer cells (69), and glioblastoma stem cells (70) through the up-regulation of markers associated with the cell cycle, angiogenesis, and proliferation. In a similar study, isoflurane-induced upregulation of HIF-1α, consequently increasing tumor malignancy with increased proliferation and migration, as well as the development of chemoresistance in prostate cancer cells (67). In a rat model of pulmonary metastasis, ketamine, thiopental, and halothane inhibited NK activity and promoted tumor metastasis (35). On the other hand, propofol mitigates malignant effects such as epithelial-mesenchymal transition (EMT) and HIF-1α effects (71), postpones colorectal cancer development through circ_0026344/miR-645/Akt/mTOR signal pathway (72), and inhibits the proliferation, migration, and stem-like properties of bladder cancer by suppressing the hedgehog pathway (73).

The local anesthetics, lidocaine, and ropivacaine decrease the viability and proliferation of cancer cells and increase their apoptosis. Mechanistically, lidocaine upregulates the mRNA level of adenomatous polyposis coli, which serves as an inhibitor of the Wnt/β-catenin pathway, while ropivacaine reduces the mRNA level of important cell cycle modulators such as cyclin A2, cyclin B1, cyclin B2, cyclin-dependent kinase 1, and the nuclear marker of cell proliferation MKI67 (74). Lidocaine inhibits the growth of hepatocellular carcinoma cells in a dose- and time-dependent manner by arresting cells in the G0/G1 phase of the cell cycle, and inducing apoptosis. It suppressed tumor development and improved the sensitivity of cisplatin (75). In another study, during sevoflurane anesthesia, the addition of lidocaine to cisplatin significantly reduced metastatic lung but not liver colony count compared to sevoflurane alone and cisplatin alone. Additionally, serum interleukin-6 and VEGF levels were not significantly different (76). This indicates that under sevoflurane anesthesia, lidocaine capably enhances the metastasis-inhibiting function of cisplatin in a murine model of breast cancer surgery. Moreover, mice that receive lidocaine with sevoflurane exhibit reduced lung metastatic colony count, as well as decreased serum pro-inflammatory and angiogenic cytokine expression (77).

Metastatic colon and breast cancer cells express adult and neonatal splice variants of NaV1.5 voltage-activated Na(+) channels. Blockade of these channels inhibits cell invasion. Local anesthetics employed during surgical tumor excision inhibit NaV1.5 voltage-activated Na(+) channels activity on nociceptive neurons, providing regional anesthesia (78, 79). Ropivacaine inhibits both NaV1.5 channel activity and metastatic colon cancer cell invasion (80). Moreover, lidocaine and levobupivacaine potently inhibit aNaV1.5, where higher concentrations of either levobupivacaine (100 μM) or lidocaine (300 μM) result in significantly more tonic block at -120 mV (78). These findings indicate that low concentrations of local anesthetics exhibit an inactivation-dependent block of NaV1.5, and could provide a rationale for their application to safely impede the migration and invasion of metastatic cancer cells without cardiotoxicity.

### Retrospective Studies

Several human studies, mainly retrospective, have shown different effects of anesthetics on cancer cell growth and recurrence after surgical removal. These studies mainly compare the different patient outcomes between anesthesia techniques or anesthetic agents. A systematic review of the overall mortality and post-surgery complications after tumor surgery with intravenous and inhalational anesthesia techniques reported that four propensity-adjusted retrospective studies show intravenous anesthesia to be the preferred technique in tumor surgery (81). The result of similar meta-analyses of the effects of propofol (intravenous) and volatile (inhalational gas) anesthesia on cancer recurrence and survival suggested that propofol-based total intravenous anesthesia use might be associated with enhanced recurrence-free survival and overall survival in patients having cancer surgery (82, 83). Another study found volatile inhalational anesthesia to be associated with a hazard ratio of 1.59 (1.30 to 1.95) for death on univariate analysis and 1.46 (1.29 to 1.66) after multivariable analysis of known confounders (84). This implies an association between the type of anesthetic delivered and patients’ survival. However, these pieces of evidence suffer moderate to serious risk of bias and of low quality, hence randomized clinical trials are needed for concrete confirmation of these findings.

Volatile anesthetic agents have been implicated in metastasis-enhancing effects on cancer cells. Notwithstanding, Xenon, but not sevoflurane, inhibits the migration of both estrogen receptor-negative and positive breast adenocarcinoma cells, and reduces the release of the pro-angiogenic factor RANTES (regulated upon activation, normal T Cell expressed and presumably secreted) (85). In a retrospective cohort study of patients who received elective, open pancreatic cancer surgery, the effect of anesthetic techniques (propofol vs. desflurane) on patients’ outcomes has been reported. Propofol anesthesia was associated with enhanced survival in matched analysis and significantly better cancer-specific survival in subgroup analyses. Moreover, propofol was linked with less postoperative recurrence, but not fewer postoperative metastases formation compared to desflurane (86). In a similar retrospective cohort study of colon cancer patients, propofol anesthesia had better survival than desflurane, irrespective of lower tumor-node-metastasis stage, or higher tumor-node-metastasis stage, and the presence or absence of metastases (87). Another report indicates that the five-year survival rate of patients that underwent general anesthesia during bladder tumor surgery is 87.5% compared to 96.3% for regional anesthesia. The authors conclude that although partial correlation analysis showed a higher five-year survival under regional than general anesthesia, the association was not significant in the chi-square test and logistic regression analysis (88).

However, several others studies have reported no significant difference between the type or method of anesthesia used during tumor surgery. For example, in non-randomized retrospective analysis, neither propofol nor desflurane anesthesia for breast cancer surgery exhibited any significant effect on patient prognosis and survival (89). Again, no obvious relationship was found between epidural anesthesia use and long-term survival according to the Cox model, but the Kaplan-Meier analysis showed an association among younger patients (15). A recent cohort study found no association between the type of anesthesia used (total IV anesthesia vs inhalation anesthesia) and the long-term prognosis of breast cancer after surgery (22). However, in a similar study that evaluated the influence of regional anesthesia on cancer-specific outcomes in a radical cystectomy cohort of patients, the authors concluded that epidural anesthesia using sufentanil is linked with worse recurrence and disease-free survival in bladder cancer patients treated with surgery. The cumulative risk of recurrence at two years was 25.2% for epidural analgesia with general anesthesia compared to 20.0% for general anesthesia alone. This could be due to the use of epidural sufentanil or the increased total morphine equivalents patient received as a consequence of the sufentanil (90). **Table 1** summarizes preclinical and retrospective studies concerning the outcome of various anesthetic agents on tumors.

**Table 1:** Preclinical and retrospective studies on anesthesia effects on tumor cells.

Anesthesia agent/technique	Study model	Tumor type	Outcome	Reference
Ropivacaine	SW620 cells *in vitro*	Colon	Ropivacaine causes a concentration-dependent blockade of NaV1.5 variants, inhibiting migration and invasion of metastatic cancer cells	(80)
Xenon and sevoflurane	*In vitro*	Breast	Xenon, but not sevoflurane, inhibits tumor cell migration and expression of angiogenesis biomarkers, RANTES	(85)
Lidocaine and sevoflurane	4T1 murine model (female BALB/c mice)	Breast	Under sevoflurane anesthesia, lidocaine enhances the metastasis-inhibiting action of cisplatin	(76)
Lidocaine and sevoflurane	4T1 murine model (female BALB/c mice)	Breast	Lidocaine decreases pulmonary metastasis combined with sevoflurane, perhaps *via* anti-inflammatory and anti-angiogenic effects	(77)
Lidocaine	*In vitro* and xenograft model *in vivo*	Hepatocellular (HepG2 cells)	Lidocaine exerts potent antitumor activity in hepatocellular carcinoma	(75)
Lidocaine and levobupivacaine	HEK-293 cells *in vitro*	–	Lidocaine and levobupivacaine potently inhibited aNaV1.5, inhibiting migration and invasion of metastatic cancer cells	(78)
Sevoflurane with/without bupivacaine and morphine	C57BL/6 mice	Liver	The addition of spinal block to sevoflurane general anesthesia attenuates the suppression of the tumoricidal function of liver mononuclear cells, and preserves Th1/Th2 balance, hence reducing the promotion of tumor metastasis.	(16)
Sevoflurane	*In vitro* and *in vivo* mice model	Lung	Promotes the proliferation of Lewis lung carcinoma cells *in vitro* but may not affect proliferation *in vivo*	(41)
Isoflurane	*In vitro* use of ovarian cancer (SK-OV3) cells	Ovarian	Isoflurane exposure significantly increases angiogenic markers vascular endothelial growth factor (VEGF), insulin-like growth factor (IGF)-1 and IGF-1R expression, cell cycle progression, and cell proliferation in tumor cells	(69)
Isoflurane and propofol.	*In vitro* use of prostate cancer (PC3) cell line	Prostate	Isoflurane increases tumor malignancy *via* modulation of the HIF-1α pathway	(67)
Propofol	*In vitro* and nude mice (bladder cancer stem cells)	Bladder	Blocks the activation of the Hedgehog pathway to repress the growth of cancer cells and the tumor formation	(73)
Propofol and desflurane	A retrospective cohort study in human	Pancreatic	Propofol is associated with improved survival compared with desflurane	(86)
Propofol and desflurane	A retrospective cohort study in human	Colon	Propofol is associated with better survival irrespective of tumor-node-metastasis stage	(87)
Total IV anesthesia and inhalation anesthesia	A retrospective cohort study in human	Breast	No significant difference in recurrence-free survival or overall survival between the two groups	(22)
Desflurane or propofol	Retrospective comparative study	Breast	Neither propofol nor desflurane anesthesia for breast cancer surgery by an experienced surgeon affects patient prognosis and survival	(89)
Volatile IV Anesthesia	Retrospective comparative study	Several types	There is an association between the type of anesthetic delivered and patients’ survival.	(84)
Inhalation vs intravenous anesthesia	Retrospective study	Colorectal	Inhalation anesthesia is associated with an increased risk of recurrence after colorectal cancer surgery	(91)

### Clinical Trial Studies

The largest available randomized controlled trial at 13 hospitals in Austria, Argentina, China, Ireland, Germany, New Zealand, USA, and Singapore was carried out from 2007 to 2018 and involved 2132 women with breast cancer. Participants were assigned to undergo regional anesthesia-analgesia (1043 patients) using paravertebral blocks and the anesthetic propofol and general anesthesia (1065 patients) using the volatile anesthetic sevoflurane and opioid analgesia. Results showed that 102 (10%) of patients who underwent regional anesthesia-analgesia had breast cancer recurrences compared to 111 (10%) of those allocated to general anesthesia. Moreover, incisional pain was reported by 442 (52%) of 856 patients and 239 (28%) of 854 patients in the regional anesthesia-analgesia group at 6 and 12 months respectively, compared to 456 (52%) of 872 patients and 232 (27%) of 852 patients in the general anesthesia group. Neuropathic breast pain did not also differ by the anesthetic technique used (92). Based on this study, regional anesthesia-analgesia does not decrease breast cancer recurrence after potentially curative surgery compared to general anesthesia, and the severity and frequency of persistent incisional breast pain are unaffected by the anesthetic technique employed.

Another clinical trial that assessed postoperative circulating tumor cell count in breast cancer patients to determine how anesthesia might indirectly affect prognosis has been documented. In that randomized controlled trial, 210 participants were assigned to either sevoflurane (107 patients) or propofol (103 patients) anesthesia. Results showed that anesthesia type did not affect circulating tumor cell counts over time or positivity. However, in one secondary analysis, the administration of sevoflurane was associated with a significant increase in maximal tumor cell counts postoperatively. There was no link between NK cell activity and circulating tumor cell counts (93). CD 39 and CD73, enzymes expressed on the surface of regulatory T cells, promote cancer recurrence and metastasis by suppressing immune cells. In a randomized trial, the immunosuppressive effect of propofol and volatile sevoflurane-based anesthesia, regarding CD39 and CD73 expression on regulatory T cells was examined. Results indicated no difference in CD39 and CD73 expression on regulatory T cells between the two anesthetic agents used, as well as in helper T cell type 1 (Th1), Th17, NK cells, cytotoxic T cells, cytokines, and the neutrophil-to-lymphocyte ratio (94). This study implies similar effects regarding postoperative changes in immune cells after the use of propofol and sevoflurane in cancer surgery. Another randomized trial that investigated the effect of propofol and desflurane anesthesia on the surgery-induced immune perturbation in patients undergoing breast cancer surgery reported that, although both anesthetic agents preserved the CD4(+)/CD8(+) T cell and IL-2/IL-4 ratio, the propofol group had lower leukocytes count (with a significant reduction in NK cells) than the desflurane group (95).

Several small-sized clinical trials have also been documented. These include the report that propofol/remifentanil-based total intravenous anesthesia effectively prevents the expression of VEGF-C induced by breast surgery compared to sevoflurane-based inhalational anesthesia, but appears to be non-beneficial in the short-term recurrence rate of breast cancer (24). The clinical trial studies discussed above, among others, are summarized in **Table 2**.

**Table 2:** Clinical trial studies on anesthesia and its effects on tumor cells.

Anesthesia agent/technique	Tumor type	Clinical trial-type	Key observation	Reference
Regional anesthesia-analgesia (paravertebral blocks and anesthetic propofol) and general anesthesia (sevoflurane and opioid analgesia)	Breast	Randomized controlled	Regional anesthesia-analgesia did not reduce cancer recurrence after potentially curative surgery compared with general anesthesia	(92)
Sevoflurane and propofol	Breast	Randomized controlled	No difference between sevoflurane and propofol concerning circulating tumor cell counts over time	(93)
Sevoflurane and propofol	Breast	Randomized controlled	Both induce a favorable immune response in terms of preserving IL-2/IL-4 and CD4(+)/CD8(+) T cell ratio Reduced leukocytes and NK cells in propofol anesthesia	(95)
Sevoflurane-based inhalational anesthesia and propofol/remifentanil-based total intravenous anesthesia	Breast	Randomized controlled	Propofol/remifentanil inhibit the release of VEGF-C No significant differences in the preoperative and postoperative TGF-β concentrations between the two groups	(24)
General anesthesia vs combined epidural-general anesthesia	Gallbladder	Randomized controlled	Combined epidural-general anesthesia might attenuate intraoperative hemodynamic responses and improve postoperative cellular immunity	(96)
Volatile general anesthesia or propofol general anesthesia combined with paravertebral regional anesthesia	Breast	Randomized single-blind	The anesthetic technique did not affect neutrophil extracellular trapping expression, hence not a viable marker of the effect of anesthetic technique on breast cancer recurrence.	(97)
Sevoflurane, sevoflurane plus i.v. lidocaine, propofol, and propofol plus i.v. lidocaine	Breast	Randomized controlled	Regardless of the general anesthetic technique, lidocaine decreased postoperative expression of neutrophil extracellular trapping and MMP3, hence might reduce recurrence.	(98)
General anesthesia or combined general/epidural anesthesia	Adenocarcinoma Prostate cancer	Randomized controlled	No difference was observed between the groups in disease-free survival at a median follow-up time of 4.5 years.	(99)
Intraperitoneal local anesthetic vs placebo	Colon	Randomized controlled	There was no significant difference in overall survival or all-cause mortality. There was a higher incidence of cancer-specific mortality in the local anesthetic group	(100)

## Discussion

Surgery remains a central component of treatment for patients with many types of cancer. However, it is well documented that surgery, regardless of how extensive it is applied, cannot eliminate all cancer cells from the patient. Certain anesthesia, surgical stress, and pain medications commonly given during anesthesia for cancer surgery are known to suppress body defenses. In addition to any pre-existing micro-metastases, surgical removal of tumors results in spillage of tumor cells locally and into the bloodstream and lymphatics system. Multiple peri-operative factors, inflammatory and neurohumoral factors, patient’s physiologic response to surgery, and care of the patient after the procedure, can encourage the invasiveness and proliferation of residual tumor cells while enhancing neo-angiogenesis to support the growth. Parallel to these effects on the tumor cells, the factors could also inhibit cell-mediated immunity, the body’s capability to eliminate these tumor cells, within this same vulnerable period. Therefore, surgery and anesthesia might contribute to long‐term cancer recurrence. Current laboratory experimental data show that perioperative interventions influence cancer recurrence or metastasis by affecting cancer cell signaling, immune response, and regulating the neuroendocrine stress response.

In effect, both anesthesia and surgery depress cell-mediated immunity and upregulate angiogenesis and could therefore enhance the proliferation and metastasis of tumor cells during the perioperative period. Declined levels of circulating anti-inflammatory cytokines and alterations in the function of NK cells are among the mechanisms by which anesthetic agents and techniques can influence immune function. Other studies have asserted that the use of regional analgesia, including epidural and paravertebral block, is effective in reducing inflammation and preventing immunosuppression in patients undergoing cancer surgery. However, there are reports of no significant difference between the types or methods of anesthesia used and cancer recurrence or patients’ outcomes. Unfortunately, current evidence from clinical studies is particularly limited, largely retrospective, smaller sample size, and often contradictory, causing several questions and providing few answers. Moreover, these pieces of evidence suffer moderate to serious risk of bias and of low quality, hence randomized clinical trials are needed for concrete confirmation of these findings. In the phase of the limited data in clinical trials upon which to make concrete recommendations, clinicians and anesthesiologists may seek optimal anesthesia and analgesia for their cancer patients based on the best available evidence on outcomes and individual risk-benefit analysis.

## Conclusion

Available evidence from experimental cell culture and animal model studies, as well as clinical retrospective studies, indicate that current data are sufficient only to generate a hypothesis that anesthetic or analgesic agents contribute to cancer recurrence and metastasis. Moreover, recent randomized controlled clinical trials, including the largest study (NCT00418457), report no difference in cancer recurrence between regional and general anesthesia after potentially curative surgery. Again, the severity and frequency of persistent incisional pain are unaffected by the anesthetic technique. Until further evidence strongly implicates anesthesia in clinical trials, clinicians may continue to choose the optimum anesthetic-analgesic agents and techniques in consultation with their cancer patients, based on their expertise and current best practice.

## Author Contributions

XL designed the study and participated in manuscript writing. QW constructed the tables and figures and revised the manuscript. All authors contributed to the article and approved the submitted version.

## Conflict of Interest

The authors declare that the research was conducted in the absence of any commercial or financial relationships that could be construed as a potential conflict of interest.

## Publisher’s Note

All claims expressed in this article are solely those of the authors and do not necessarily represent those of their affiliated organizations, or those of the publisher, the editors and the reviewers. Any product that may be evaluated in this article, or claim that may be made by its manufacturer, is not guaranteed or endorsed by the publisher.

## Abbreviations

NK cells, natural killer cells; DCs, dendritic cells; MOR, µ-opioid receptor; SP, substance P; VEGF, vascular endothelial growth factor; HIF-1α, hypoxia-inducible factor-1; EMT, epithelial-mesenchymal transition.
